# Evaluation of the Cardiotoxicity of Evodiamine In Vitro and In Vivo

**DOI:** 10.3390/molecules22060943

**Published:** 2017-06-09

**Authors:** Weifeng Yang, Lina Ma, Sidi Li, Kaiyu Cui, Lei Lei, Zuguang Ye

**Affiliations:** 1 Institute of Chinese Materia Medica, China Academy of Chinese Medical Sciences, Beijing 100700, China; malina8512@163.com (L.M.); lisidimaa@aliyun.com (S.L.); 2 Experimental Research Center, China Academy of Chinese Medical Sciences, Beijing 100700, China; sunzhuyang@126.com; 3 Pharmacy Faculty, Hubei University of Chinese Medicine, Wuhan 430065, China; cky1555@sina.com; 4 Institute of Information on TCM, China Academy of Chinese Medical Sciences, Beijing 100700, China; sophlei@hotmail.com

**Keywords:** evodiamine, primary cultured neonatal rat cardiomyocytes, zebrafish, cardiotoxicity, oxidative stress

## Abstract

Evodiamine is a bioactive alkaloid that is specified as a biomarker for the quality assessment of *Evodia rutaecarpa* (*E. rutaecarpa*) and for traditional Chinese medicines containing this plant. We previously reported that quantitative structure–activity modeling indicated that evodiamine may cause cardiotoxicity. However, previous investigations have indicated that evodiamine has beneficial effects in patients with cardiovascular diseases and there are no previous in vitro or in vivo reports of evodiamine-induced cardiotoxicity. The present study investigated the effects of evodiamine on primary cultured neonatal rat cardiomyocytes in vitro, and on zebrafish in vivo. Cell viability was reduced in vitro, where evodiamine had a 24 h 50% inhibitory concentration of 28.44 µg/mL. Cells exposed to evodiamine also showed increased lactate dehydrogenase release and maleic dialdehyde levels, and reduced superoxide dismutase activity. In vivo, evodiamine had a 10% lethal concentration of 354 ng/mL and induced cardiac malfunction, as evidenced by changes in heart rate and circulation, and pericardial malformations. This study indicated that evodiamine could cause cardiovascular side effects involving oxidative stress. These findings suggest that cardiac function should be monitored in patients receiving preparations containing evodiamine.

## 1. Introduction

Computational toxicology aims to complement other measures of toxicity by helping to predict toxicity, prioritize chemicals, guide toxicity tests, and minimize late-stage failures in drug development [1]. A range of in silico methods have been developed to predict the toxicity of chemicals. Quantitative structure-activity relationship (QSAR) models are widely used for the early prediction of potential toxic effects. This approach assumes that chemicals with similar structural features operate via similar mechanisms [2]. Traditional Chinese herbs are widely used to produce important preparations employed in Oriental medicine. Although the therapeutic efficacy of this type of medicine is supported by clinical observations, it still lacks a formal evidence base relating to the pharmacodynamic effects and potential toxicity. Further studies are therefore urgently required to determine the toxicity of traditional Chinese herbal preparations. As traditional Chinese medicines contain multiple chemical components, toxicity prediction is also challenging. We previously established a QSAR model to predict the cardiotoxicity of chemical components of Chinese herbs. Using this model, analysis of the chemical components of Chinese herbs recorded in the Pharmacopoeia of the People’s Republic of China (version 2010) identified some herbs that contained significant proportions of potentially cardiotoxic compounds; these included *Evodia rutaecarpa*, *Menispermum dauricum* DC, and *Murraya exotica* L. [3].

The present study focuses on this prediction of a cardiotoxic effect of a major bioactive alkaloid present in *Evodia rutaecarpa* (*E. rutaecarpa*), evodiamine. Preparations containing this plant, which is known as Wu-Zhu-Yu, have been prescribed for the treatment of a range of conditions, including abdominal pain, headache, menstrual problems, vomiting and diarrhea [4]. *Evodia rutaecarpa* was noted as an herb with mild toxicity in “Shen Nong’s Herbal Classic”, China’s most ancient herbal medicine book. Alkaloids such as evodiamine, rutaecarpine, dihydroevocarpine and evodine are the main bioactive ingredients of *Evodia rutaecarpa* [5]. Evodiamine is specified as a biomarker for the quality assessment of *Evodia rutaecarpa*, and of traditional Chinese medicines containing this plant, in the Chinese Pharmacopoeia. This major bioactive alkaloid has a wide range of bioactivities, which include antinociceptive, anti-obesity, antitumor, vasodilatory and anti-inflammatory effects [6]. Previous investigations have indicated that evodiamine has a beneficial effect in cardiovascular diseases [7,8,9] and there are no previous reports of evodiamine-induced cardiotoxicity, either in vitro or in vivo. The present study investigated the cardiotoxicity of evodiamine on primary cultured neonatal rat cardiomyocytes in vitro, and on zebrafish in vivo.

## 2. Results

### 2.1. In Vitro Cardiotoxicity in Primary Neonatal Rat Cardiomyocytes

#### 2.1.1. Evodiamine-Induced Changes in Cardiomyocyte Viability

**Figure 1:**
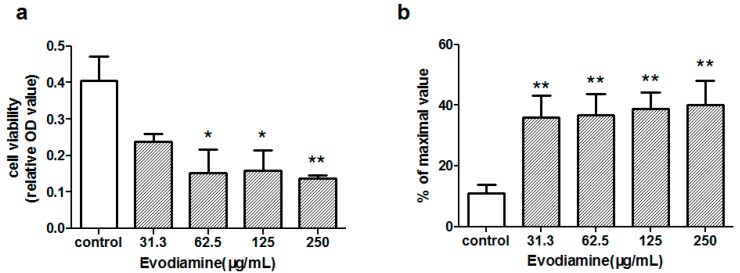
Evodiamine-induced cell injury in primary neonatal rat cardiomyocytes. Cardiomyocytes were exposed to the indicated concentrations of evodiamine for 24 h. Cell viability was measured by (**a**) cell counting kit-8 (CCK-8) assay and (**b**) lactate dehydrogenase (LDH) release assay. Data were presented as the mean ± standard deviation (SD) of three independently prepared samples, each with 3 measurements. * *p* < 0.05 and ** *p* < 0.01, compared with the control group; OD, optical density.

The effects of evodiamine on cell viability were assessed using a cell counting kit-8 (CCK-8) assay and a lactate dehydrogenase (LDH) release assay. Primary cardiomyocytes were exposed to 31.3, 62.5, 125 or 250 µg/mL evodiamine for 24 h. The CCK-8 assay results presented in Figure 1a showed that exposure to >62.5 µg/mL evodiamine significantly decreased the numbers of viable cells (*p* < 0.05), with a 50% inhibitory concentration of 28.44 µg/mL. The extent of cellular injury was also monitored by measuring LDH release. As compared with the control cells, LDH release was increased in cells exposed to >31.3 µg/mL evodiamine for 24 h (*p* < 0.01; Figure 1b).

#### 2.1.2. Evodiamine-Induced Changes in Maleic Dialdehyde (MDA) Levels and Superoxide Dismutase (SOD) Activity

**Figure 2:**
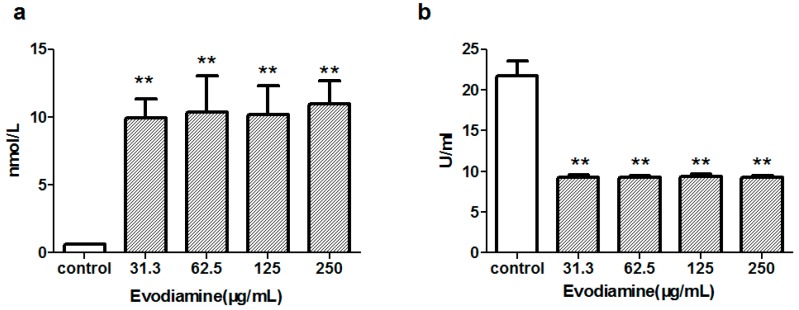
Evodiamine-induced oxidative stress in primary neonatal rat cardiomyocytes. Cardiomyocytes were exposed to the indicated concentrations of evodiamine for 24 h. (**a**) Maleic Dialdehyde (MDA) activity; (**b**) Superoxide Dismutase (SOD) activity. Data were presented as the mean ± standard deviation of three independently prepared samples, each with 3 measurements. ** *p* < 0.01, compared with the control group.

MDA levels were evaluated to provide an estimate of the degree of lipid peroxidation. SOD, an important antioxidant enzyme, plays a pivotal role in preventing the cellular damage caused by reactive oxygen species (ROS). To assess whether evodiamine-induced cardiomyocyte injury involved oxidative stress, the levels of MDA and activity of SOD were measured in cells exposed to evodiamine for 24 h. As shown in Figure 2, cells exposed to 31.3–250 μg/mL evodiamine for 24 h showed significantly increased MDA levels, while they showed significantly decreased SOD activity.

### 2.2. In Vivo Cardiotoxicity in Zebrafish

#### 2.2.1. Determination of the Maximum Non-Lethal Concentration (MNLC) and 10% Lethal Concentration (LC_10_)

**Figure 3:**
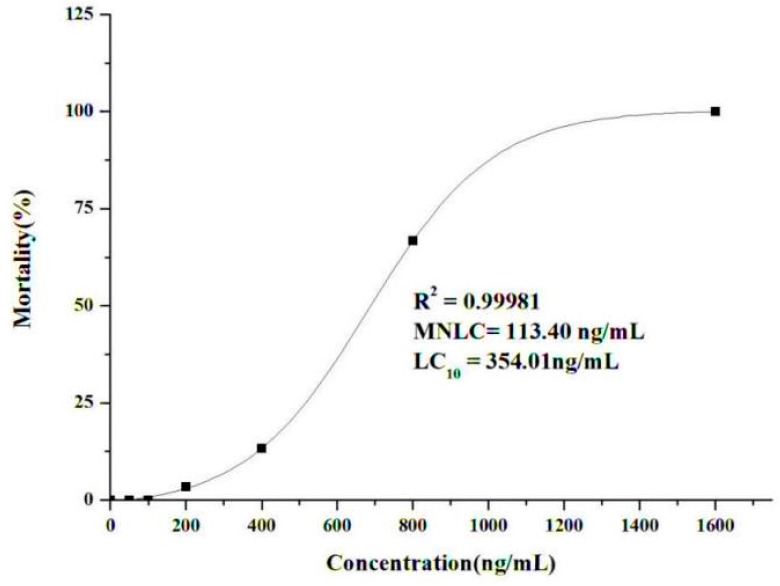
The effects of evodiamine on zebrafish mortality. Zebrafish were exposed to evodiamine at the indicated concentrations for 24 h. All data are represented as the mean ± standard deviation; *n* = 30 zebrafish for each concentration.

An increase in wild-type AB zebrafish lethality was observed following exposure to different concentrations of evodiamine for 24 h (Figure 3). No lethal effect was observed in the presence of 50–100 ng/mL evodiamine, while there was a sharp increase in lethality at concentrations ≥400 ng/mL; lethality reached 100% at 1600 ng/mL evodiamine. The MNLC and LC_10_ values were estimated as 113.4 ng/mL and 354 ng/mL, respectively, using sigmoidal regression in Origin 8.0 software. Based on these findings, cardiotoxicity assessments were conducted in zebrafish exposed to one-tenth of the MNLC (11 ng/mL), one-third of the MNLC (38 ng/mL), the MNLC (113 ng/mL), and the LC_10_ (354 ng/mL).

#### 2.2.2. Evodiamine-Induced Effects on Heart Rate and Rhythm

**Figure 4:**
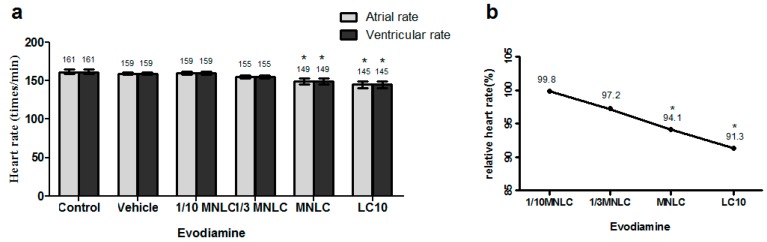
The effects of evodiamine on the zebrafish heart rate. Heart rate is shown as the absolute rate (**a**) and the relative rate (**b**), expressed as a % of the heart rate in the vehicle control group. All data are represented as the mean ± standard deviation; *n* = 10; * *p* < 0.001, compared with the vehicle control group.

The data presented in Figure 4a indicate that the atrial and ventricular heart rates were decreased in a dose-dependent manner in wild-type AB zebrafish exposed to evodiamine. The heart rates were 149 ± 2.0 and 145 ± 2.4 beats/min in zebrafish exposed to the MNLC and LC_10_ of evodiamine, respectively, as compared to the vehicle controls (159 ± 0.9 beats/min; *p* < 0.001 for both). These corresponded to decreases to 94.1% and 91.3% of the vehicle control value (Figure 4b). Heart rhythm assessment revealed no differences between the atrial and ventricular rates.

#### 2.2.3. Morphological Assessment of Cardiotoxicity

**Figure 5:**
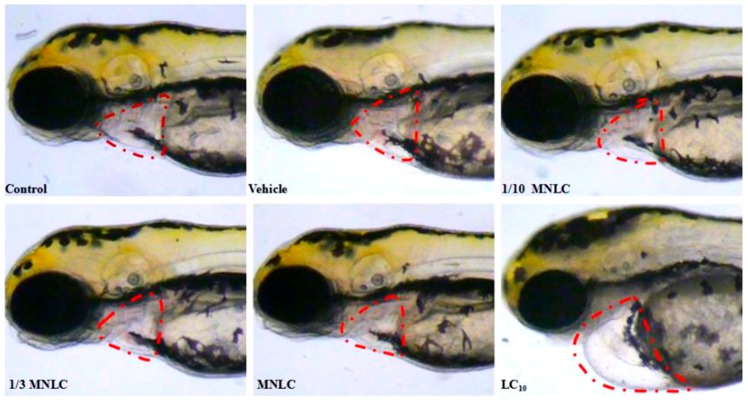
Visual observation of zebrafish larvae after exposure to the indicated concentrations evodiamine for 24 h. The circled area indicates the zebrafish heart and pericardium.

Cardiovascular toxicity-associated morphological abnormalities were observed and assessed quantitatively; these included heart malformation, pericardial edema, circulation abnormalities, thrombosis and hemorrhage. As shown in Figure 5, exposure to the LC10 of evodiamine (354 ng/mL) was associated with pericardial edema (observed in 17/28 zebrafish), reductions in blood circulation (observed in 10/28 zebrafish), or loss of circulation (observed in 9/28 zebrafish, 2/30 zebrafish were dead, and 28 zebrafish were actually observed). These changes were not observed in zebrafish exposed to lower levels of evodiamine.

**Figure 6:**
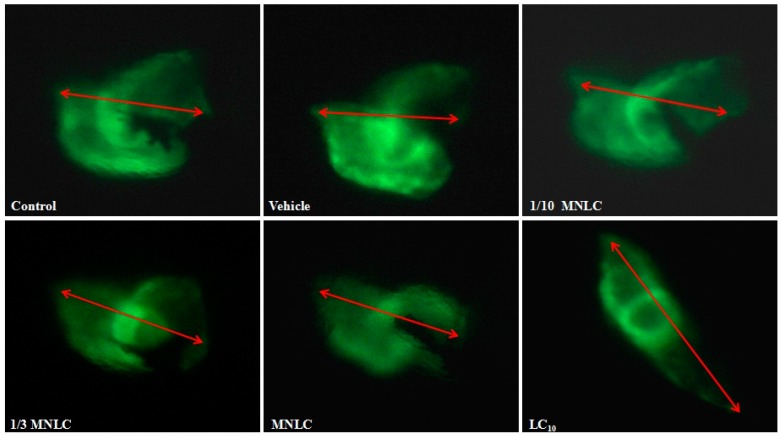
The measurement of SV–BA distance following exposure to evodiamine for 24 h. The arrows indicate the direct distance between the centers of the SV and BA.

The distance between the sinus venosus (SV) and bulbus arteriosus (BA) provides a marker for the development of the heart into two distinct chambers [10]. To examine the effects of evodiamine on cardiac development, the SV–BA distance was measured in cardiac myosin light chain 2 (*cmlc2*) transgenic zebrafish, which express enhanced green fluorescent protein (GFP) in the heart. This facilitated direct observation of the heart using fluorescent imaging. As indicated by the data shown in Table 1 and Figure 6, the SV–BA and the relative (%) SV–BA distances were significantly increased in zebrafish exposed to 38 ng/mL, 113 ng/mL, or 354 ng/mL evodiamine, as compared to the vehicle group. 

## 3. Discussion

Previous studies have mainly focused on the hepatotoxicity of *E. rutaecarpa* because prescription of high levels of this preparation containing this plant has been reported to cause hepatotoxicity in humans [11]. *E. rutaecarpa* aqueous extract produces obvious cumulative toxic effects on the liver [12,13]. Water-extracted components may induce acute hepatic injury, which shows some dosage- and time-dependency [14,15]. The relationships between the constituents of *E. rutaecarpa* and hepatotoxicity remain unclear because the toxic effects of this plant and of evodiamine have not been fully evaluated. Our previous study established a QSAR model to predict the cardiotoxicity of chemical components of Chinese herbs [3]. This model indicated that *E. rutaecarpa* and evodiamine may have a high risk of inducing cardiac toxicity. Even though computational methods cannot substitute for biological testing, they have the unique advantage of being able to estimate chemical toxicity and can thus improve efficiency by prioritizing the testing of particular substances and targets [1]. 

The present study initially tested the cardiac risk associated with evodiamine using primary cultured neonatal rat cardiomyocytes in vitro. This study showed that exposure to evodiamine reduced the number of viable cells, with a 24 h 50% inhibitory concentration of 28.44 µg/mL, and increased LDH release (Figure 1). These in vitro results were consistent with the proposal that evodiamine can cause cardiotoxicity. Cai et al. demonstrated that an *E. rutaecarpa* extract induced oxidative damage in rat mitochondria, leading to ATP (Adenosine Triphosphate) depletion and cytochrome c release, which triggered cell death signaling pathways; these are the mechanisms underlying the hepatotoxicity of *E. rutaecarpa* [15]. The present observations of evodiamine-induced changes in MDA levels and SOD activity also indicated that oxidative stress may play an important role in the cardiotoxicity of evodiamine (Figure 2).

Cardiotoxicity was also evaluated in vivo using a wild-type zebrafish (AB) strain and a *cmlc2* EGFP (Enhanced Green Fluorescent Protein) transgenic zebrafish. The zebrafish provides an extremely useful in vivo model in which to study the effects of drugs or toxic substances on vertebrate development, and particularly on cardiac development [16,17]. A previous report found that the overall predictive success rate of zebrafish for cardiotoxicity was 100% [18]. Our results indicated that exposure to 354 ng/mL evodiamine for 24 h induced marked cardiac changes (Figure 5), while concentrations of ≥38 ng/mL affected cardiac development (Figure 6). No morphological changes were found in the heart or pericardium in the vehicle control fish.

Recent studies have also reported that evodiamine showed antitumor activity on various human cancer cells [19,20,21,22,23,24,25,26], although the effective dose of this compound had weaker cytotoxic effects than actinomycin D or fluorouracil on normal human peripheral blood cells or on the body weight of tumor-bearing mice [27]. Evodiamine has therefore attracted the interest of pharmacologists as a potential drug in the field of oncology. However, many antitumor drugs are known to cause cardiotoxicity during the treatment period, or to introduce a measurable increase in the risk of delayed cardiovascular events [28,29]. This risk of cardiotoxicity increases in patients with hypertension, diabetes mellitus, liver disease and previous cardiac diseases [30]. The redox environment of the cell is extremely important to control either apoptosis or autophagy. One of the most widely cited and accepted mechanisms underlying cardiotoxicity involves the formation of ROS, leading to oxidative stress [31,32,33,34]. A previous study reported that ROS and nitric oxide (NO) generation was induced by evodiamine time-dependently in human cervical carcinoma HeLa cells [25]. Evodiamine can also influence the G2/M cell cycle by inducing ROS/NO generation [26]. These studies indicate that ROS and NO play pivotal roles in mediating the cytotoxicity of evodiamine. The present findings and the previous reports of *E. rutaecarpa*-induced hepatotoxicity indicate that evodiamine may produce adverse effects when used as an anti-cancer agent. Even though data generated using laboratory animal models do not always predict the mechanisms and/or metabolic determinants of cardiotoxicity in humans, the zebrafish offers numerous advantages for toxicological research that are not found in other model systems. This may explain why no previous reports have identified the cardiotoxic effects of evodiamine.

As we all know, adverse cardiac effects are also the leading cause of drug discontinuation and failure of clinical trials. Cardiotoxicity accounted for 45% of all drugs withdrawn between 1994 and 2006, which was due mainly to cardiac ischemia-related and arrhythmogenic side effects [35]. Withdrawal of a cardiotoxic drug is not just a waste of time and money for the pharmaceutical industry, but may also impose serious risks to a patient’s life. Therefore, predicting the side effects of a drug at an early stage of its development is of utmost importance as regards drug safety assessments [36]. To predict the safety of a drug at an early stage in its development is a major challenge as there is a lack of in vitro heart models that correlate data from preclinical toxicity screening assays with clinical results [37]. Computer toxicology, which has developed rapidly over the past few decades, provides a method to predicting drug toxicity. However, computational toxicity studies of chemical compounds used as drugs have just begun [38] and there are few reports describing its application to traditional Chinese medicines. The present study verified the results of a QSAR model [2] using primary cultured neonatal rat cardiomyocytes in vitro and zebrafish in vivo. Both of these systems indicated that evodiamine may cause cardiovascular side effects, driven by oxidative stress. These findings could form the basis for further research into this potential effect of evodiamine and also provide an approach to evaluating the toxicity of other traditional Chinese medicines.

## 4. Materials and Methods 

### 4.1. Chemicals, Drugs and Reagents

Evodiamine (CAS No. 518-17-2) was obtained from Weikeqi Biological Technology Co., Ltd. (Chengdu, Sichuan, China). The purity of evodiamine was measured by high-performance liquid chromatography (HPLC) and determined to be about 98%.

Dulbecco’s modified Eagle’s medium/F-12 (DMEM/F-12), fetal bovine serum (FBS), penicillin-streptomycin-glutamine (×100), and trypsin were purchased from Thermo Fisher Scientific Co., (Carlsbad, CA, USA). Dimethyl sulfoxide (DMSO; tissue culture grade) was purchased from Sigma-Aldrich Inc. (St. Louis, MO, USA). The Cell Counting Kit-8 (CCK-8) assay was purchased from Dojindo (Tokyo, Japan), and LDH, SOD and MDA were purchased from Jiancheng Bioengineering Institute, Nanjing, China.

### 4.2. Animals

Sprague-Dawley rats (1–3 days old) were purchased from Beijing Vital River Laboratory Animal Technology Co., Ltd. All animals used in this study were cared for in accordance with the Guide for the Care and Use of Laboratory Animals, which was published by the United States National Institutes of Health (NIH publication no. 85–23, revised 1996), and all procedures were approved by the Committee of Experimental Animals at the Institute of Chinese Meteria Medica, China Academy of Chinese Medical Sciences (Beijing, China).

Zebrafish were purchased from Hangzhou Hunter Biotechnology Co. Ltd., which was accredited by the Association for Assessment and Accreditation of Laboratory Animal Care International. The wild-type AB strain of zebrafish was used to assess acute toxicity and cardiotoxicity, while transgenic *cmlc2* zebrafish expressing EGFP were used to examine the SV–BA distance. The zebrafish were housed in a light- and temperature-controlled aquaculture facility with a standard 14:10 h day/night photoperiod. They were fed with live brine shrimp twice daily and with dry flake once daily. Zebrafish were maintained at 28 °C in fish water (0.2% Instant Ocean Salt in deionized water; pH 6.9–7.2; conductivity 480–510 μS/cm and hardness 53.7–71.6 mg/L CaCO_3_).

### 4.3. In Vitro Cardiotoxicity in Primary Neonatal Rat Cardiomyocytes

#### 4.3.1. Experimental Protocol

Evodiamine was diluted using DMSO. The concentration of DMSO in all treated cell cultures was kept below 0.1%, a level that had no detectable effect on cell growth. The control cells were treated with Dulbecco’s modified Eagle’s medium/F12 containing 0.1% DMSO.

#### 4.3.2. Isolation and Culture of Primary Neonatal Rat Cardiomyocytes

Primary neonatal rat cardiomyocytes were isolated enzymatically from 1–3-day-old Sprague-Dawley rats. The rat hearts were removed and placed in phosphate-buffered saline. The ventricles were then minced into pieces of approximately 1 mm^3^. The tissue fragments were dissociated by treatment with 0.125% trypsin 4–5 times at 37 °C, filtered, centrifuged for 10 min (1000 rpm), and resuspended in Dulbecco’s modified Eagle’s medium/F12 containing 10% fetal bovine serum, penicillin (100 U/mL), and streptomycin (100 μg/mL). The cells were then plated in a petri dish and placed in a humidified incubator (5% CO_2_, 37 °C) for 1.5–2 h to reduce fibroblast contamination. The cardiomyocytes were seeded in 96-well or 6-well plates and incubated for 3–4 days before the experiments.

#### 4.3.3. Cell Viability Assay

Cell viability was assessed by the CCK-8 assay (Dojindo, Tokyo, Japan) using the experimental procedure provided by the manufacturer. The cardiomyocytes were seeded in 96-well plates at 1 × 10^5^ cells/well and incubated with the indicated concentrations of evodiamine for 24 h; the negative control experiment (0.1% DMSO) was performed in parallel. After these treatments, 10 µL of CCK-8 was added to each well, and the cardiomyocytes were incubated for an additional 2 h at 37 °C. The absorbance of each well at 450 nm was measured using a microplate reader (iMark, BioRad, Veenendaal, The Netherlands). The following equation was used to measure cell viability: cytotoxic index = (1 − treatment group absorbance/control group absorbance) × 100%. The 50% inhibitory concentration values were determined from the dose-response plots using linear regression. Each of the experiments was performed at least three times.

#### 4.3.4. Determination of LDH Release, MDA and SOD Activity

LDH release, MDA levels and SOD activity were determined using commercially available kits (Jiancheng Bioengineering Institute, Nanjing, China), according to the manufacturer’s instructions. Cells were seeded and treated as described in Section 4.3.3. Released LDH was expressed as the level of LDH in the medium, in relation to the total cellular LDH.

### 4.4. In Vivo Cardiotoxicity in Zebrafish

#### 4.4.1. Experimental Protocol

Evodiamine was diluted using DMSO to prepare a stock solution and working solutions were prepared by dilution with fish water; the final DMSO concentration was kept below 0.01%. Zebrafish were exposed to the indicated concentrations of evodiamine in 6-well plates, containing 30 fish per dish. The temperature was 28 °C during the exposure to evodiamine and the endpoints were observed at 48 h post-fertilization. The control group was treated with fish water, and the vehicle group was treated with fish water containing 0.01% DMSO.

#### 4.4.2. Determination of the MNLC and LC_10_

The MNLC and LC_10_ of evodiamine were determined using zebrafish larvae at 48 h post-fertilization. Mortality was recorded over 24 h in the presence of the indicated concentrations of evodiamine. The mortality curve was generated using Origin 8.0 (OriginLab, Northampton, MA, USA) and MNLC and LC_10_ were determined using logistic regression.

#### 4.4.3. Cardiovascular Toxicity Assessment

Four concentrations were selected for the assessment of the cardiovascular toxicity of evodiamine in the wild-type AB zebrafish strain: one-tenth the MNLC (11 ng/mL), one-third the MNLC (38 ng/mL), the MNLC (113 ng/mL), and the LC_10_ (354 ng/mL). Following the treatment of zebrafish (72–120 h post-fertilization), 10 individuals from each group were randomly selected for visual observation and image acquisition of specific phenotypic endpoints under the dissecting stereomicroscope. The occurrence of pericardial edema, abnormal circulation (decrease or absence), thrombosis and hemorrhage were thereby evaluated by two independent observers who were blind to the study group. The heart rate (atrial and ventricular) and rhythm were determined. The relative heart rate corresponded to the heart rate of each treatment group, expressed as a percentage of the heart rate of the vehicle group.

#### 4.4.4. Morphological Assessment

The SV–BA distance was determined in transgenic zebrafish, which expressed EGFP driven by the *cmlc2* promoter. The zebrafish were treated as described in Section 4.4.3 and 10 individuals from each group were randomly selected for visual observation and imaging using a fluorescent microscope (Nikon, Japan). The SV–BA distance was the length of a straight line connecting the centers of the two structures, using a consistent magnification (Figure 6). The relative SV–BA distance corresponded to the SV–BA distance in each treatment group, expressed as a percentage of the SV–BA distance in the vehicle group.

### 4.5. Statistical Analysis

Data are expressed as the mean ± the standard deviation. All analyses were performed by one-way analysis of variance (ANOVA) using the Statistical Package for Social Sciences for windows (version 15, SPSS Inc., Chicago, IL, USA). The Duncan test for multiple comparisons was carried out to compare the mean value of the control group with that of each experimental group. Differences were regarded as significant at *p* < 0.05. 

## 5. Conclusions

The present in vitro and in vivo studies indicated that evodiamine could cause cardiovascular side effects, including reductions in circulation and pericardial malformations, and that oxidative stress plays an important role in this evodiamine-induced cardiotoxicity. These findings indicate that this potential adverse effect of evodiamine should be considered when it is administered to patients.

## Figures and Tables

**Table 1:** Zebrafish SV–BA distance changes induced by evodiamine.

Group	Evodiamine (ng/mL)	SV–BA Distance (Pixels)	Relative SV–BA Distance (%)
Control group	/	231 ± 6.6	101
Vehicle group	/	229 ± 3.5	/
1/10 MNLC	11	235 ± 2.4	102
1/3 MNLC	38	250 ± 5.1 *	109 *
MNLC	113	254 ± 7.0 *	111 *
LC10	354	326 ± 6.0 **	142 **

Data represent the mean ± standard deviation, *n* = 10; * *p* < 0.05, ** *p* < 0.001, compared with the vehicle control group. SV = sinus venosus; BA = bulbus arteriosus; MNLC = Maximum Non-Lethal Concentration.
